# Factors influencing the mental well‐being of professional guardians in Japan: The role of stress and stigma

**DOI:** 10.1002/pcn5.70114

**Published:** 2025-05-07

**Authors:** Kae Ito, Tsuyoshi Okamura

**Affiliations:** ^1^ Tokyo Metropolitan Institute for Geriatrics and Gerontology Tokyo Japan

**Keywords:** adult guardianship, mental well‐being, professional guardian, psychological stress, stigma

## Abstract

**Aim:**

As Japan increasingly relies on professional adult guardians, understanding their challenges and identifying support measures for their mental well‐being is essential. This study explores factors influencing the mental well‐being of professional guardians.

**Methods:**

Judicial scriveners, who handle 35.9% of all guardianships, represent the largest group of professional guardians, most of whom are affiliated with the Adult Guardianship Center Legal Support. An online questionnaire survey was conducted among members of the Tokyo Branch. Of the 1627 members contacted, 227 (14.0%) responded between October 2 and 23, 2024.

**Results:**

The prevalence of poor mental well‐being (World Health Organization‐Five Well‐Being Index Japanese version <13) among professional guardians was 42.3%. Univariate analysis identified factors significantly associated with poor mental well‐being, including low satisfaction, frequent psychological stress, inadequate job support, perceived poor physical health, loneliness, burnout, and community stigma. Multivariate analysis confirmed that frequent psychological stress (95% confidence interval [CI]: 0.08–0.37, *p* = 0.002) and strong community stigma (95% CI: −0.03 to 0.00, *p* = 0.043) were independently associated with poor mental well‐being.

**Conclusion:**

This study highlights significant challenges to the mental well‐being of professional guardians in Japan, particularly due to psychological stress and community stigma. Given that 42.3% of participants reported poor mental well‐being, a rate significantly higher than the general population, there is an urgent need for targeted support measures, such as stress‐management programs and peer support networks.

## INTRODUCTION

Public advocacy and awareness regarding individuals with mental health conditions in Japan are relatively recent developments. Although the Basic Act for Persons with Disabilities^1^ was enacted in 1970, individuals with mental health conditions were not explicitly addressed until a 1993 amendment. A further amendment in 2011 introduced the concept of an *inclusive society*, marking a shift from Japan's long history of institutionalization toward community‐based care. This transition was reinforced by the 2012 amendment to the Act on Comprehensive Support for the Daily and Social Life of Persons with Disabilities,^2^ which emphasizes independent living and community integration. These legislative changes laid the foundation for Japan's ratification of the United Nations Convention on the Rights of Persons with Disabilities^3^ in 2014.

Persons with mental health conditions face a high risk of human rights violations, highlighting the need for protective systems.^4^ Historically, guardianship was based on a paternalistic model dating back to Roman times. However, since the 1970s, deinstitutionalization and evolving disability rights movements have shifted guardianship philosophy toward prioritizing individual autonomy and due process.^5^

In Japan, the adult guardianship system, established under the Civil Code,^6^ grants guardians broad legal authority, including comprehensive representation, contract rescission, and the obligation to manage personal affairs.^7^ Guardians are increasingly expected to manage in service contracts, secure accommodation, arrange hospital admissions, and even make medical decisions—issues also debated in Western countries.^8^, ^9^, ^10^ However, Japanese law does not explicitly define the legal authority of guardians or other parties to make medical decisions for adults, creating significant legal and ethical ambiguities.

When the adult guardianship system was introduced in 2000, 90.9% of guardians were family members. However, as the number of older adults without available family members increased, the proportion of family guardians steadily declined to 18.1% by 2023. Conversely, professional guardians, such as lawyers, judicial scriveners, and social workers, now handle 81.9% of cases, compared to just 8.2% in 2000.^11^ While the system aims to protect individuals with reduced mental capacities, it presents significant challenges. Elements of the earlier paternalistic system persist, prioritizing family interests over individual rights and often restricting personal autonomy. The United Nations Committee on the Rights of Persons with Disabilities has criticized Japan's guardianship system for violating Article 12 of the Convention on the Rights of Persons with Disabilities.^3^

Despite calls for reform, Japan lacks a viable alternative framework. As a result, professional guardians often face complex ethical dilemmas, including balancing legal responsibilities with the rights of individuals under guardianship, handling conflicts with families (particularly in cases of financial exploitation), and navigating unclear role expectations.^12^ Beyond legal representation, they may act as accountants, legal advocates, or surrogate family members,^9^ leading to role confusion and increased burden.^13^

Given the increasing reliance on professional guardians in Japan, it is essential to understand their burden and identify measures to address it. We conducted an online questionnaire survey to explore factors associated with the mental well‐being of professional guardians.

## METHODS

### Study participants

Judicial scriveners represented the largest group of professional guardians, accounting for 35.9% of cases.^11^ Most were affiliated with the Adult Guardianship Center Legal Support. Data were collected through an online questionnaire survey targeting members of the Tokyo Branch of this organization. Out of 1627 members contacted, 227 (14.0%) responded between October 2 and 23, 2024.

### Measures

#### Main outcome

Mental well‐being was assessed using the Japanese version of the World Health Organization‐Five Well‐Being Index (WHO‐5‐J),^14^, ^15^ a standardized self‐report instrument designed to measure mental well‐being.^16^ The internal and external validity have been confirmed in studies involving elderly Japanese community residents.^15^ It comprises five statements reflecting experiences over the past 2 weeks: “I have felt cheerful and in good spirits”; “I have felt calm and relaxed”; “I have felt active and vigorous”; “I woke up feeling fresh and rested”; and “My daily life has been filled with things that interest me.” Each statement was rated on a six‐point scale (0–5), with higher scores indicating better mental well‐being. The total score ranges from 0 to 25, with the standard cutoff criterion defining poor mental well‐being as a total score of <13.

#### Covariates

##### Demographic characteristics

The collected data included the participants' age, sex, and workplace type (private office or other).

##### Professional background and job conditions

Data were collected on participants' years of experience as adult guardians, the number of adult wards currently overseen, and their experience managing adult wards diagnosed with schizophrenia, dementia, and intellectual disabilities. Years of experience were categorized as ≤3 years and >3 years, while the number of adult wards currently overseen was classified as ≤10 and ≥11.

Additionally, participants reported the percentage of total work time devoted to adult guardianship services and the proportion of total compensation derived from guardianship fees.

###### Satisfaction with adult guardianship

Participants rated their satisfaction on a scale from 1 (*very satisfied*), 2 (*fairly satisfied*), 3 (*not so satisfied*), and 4 (*not at all satisfied*). Responses of 1 and 2 were categorized as 1 (satisfied), whereas responses of 3 and 4 were categorized as 0 (not satisfied).

###### Perceived appropriateness of compensation

Participants were asked whether they felt that their compensation for adult guardianship was appropriate for their work. Possible responses were 1 (*very appropriate*), 2 (*fairly appropriate*), 3 (*not very appropriate*), and 4 (*not at all appropriate*). Responses of 1 and 2 were categorized as 1 (*appropriate*), whereas responses of 3 and 4 were categorized as 0 (*not appropriate*).

###### Psychological stress

Psychological stress related to serving as an adult guardian was assessed using two questions: “How often do you feel stressed while serving as an adult guardian?” and “What is the main source of psychological stress experienced when serving as an adult guardian?” For the first question, possible answers were 1 (*very often*), 2 (*sometimes*), 3 (*rarely*), or 4 (*not at all*). Response 1 was categorized as 1 (high frequency), while responses 2, 3, and 4 were categorized as 0 (low frequency). For the second question, participants selected their primary source of stress from the following options: 1. adult wards, 2. family members in adult wards, 3. neighbors in adult wards, 4. care managers in adult wards, 5. staff of the Comprehensive Community Support Centers, 6. staff at medical institutions, 7. administrative institutions (e.g., local authorities), and 8. judicial institutions (e.g., family courts).

###### Job support

Job support was assessed with the question, “How many people can help you when you need support concerning adult guardianship?” The possible answers were 1 (*none*), 2 (*1–4 people*), and 3 (*more than 5 people*). Response 1 was categorized as 0 (no social support), whereas responses 2 and 3 were categorized as 1 (having social support).

##### Health‐related variables

Subjective perception of physical health was assessed using the question, “How would you describe your current overall health?” Possible answers were 1 (*very good*), 2 (*fairly good*), 3 (*not good*), and 4 (*bad*). Responses 1 and 2 were categorized as 1 (good), and 3 and 4 were categorized as 0 (bad).

##### Psychosocial variables

###### Social support and loneliness

Loneliness was assessed by the Japanese version of the short form UCLA Loneliness Scale (Version 3).^17^, ^18^ The scale consists of three items, each rated by a four‐point scale (0–3), with higher scores indicating stronger loneliness. Burnout was assessed using the Japanese Burnout Scale.^19^ The scale consists of 17 items, each rated on a two‐point scale, with higher scores indicating stronger burnout.

###### Community stigma toward persons with mental health conditions

To assess guardians' perceptions of community stigma toward persons with mental health conditions, we used the Japanese version of the Link Stigma Scale.^20^, ^21^ This 12‐item scale measures how society perceives individuals with mental health conditions, with responses rated on a four‐point scale,^1^, ^2^, ^3^, ^4^ where higher scores indicate stronger perceived stigma.

### Statistical analysis

Statistical analyses were performed using SPSS Statistics Version 29.0.1.0 for Windows (IBM). The baseline characteristics of the 227 study participants were compared using simple linear regression for both continuous and categorical values, with categorical variables coded as dummy variables where necessary. The association between mental well‐being and each variable was assessed using beta coefficients (*β*), 95% confidence intervals (CIs), and *p*‐values. To explore the impact of different factors, we conducted hierarchical multiple regression analyses across four models, progressively adjusting for key variables. Model 1 included sex as a basic characteristic and years of experience as an indicator of job tenure. Model 2 incorporates the frequency of psychological stress and captures subjective job strain. Model 3 was further adjusted for the perception of compensation, representing reward‐related factors. Finally, Model 4 introduced community stigma to address the broader psychosocial influences. This stepwise approach allowed us to examine how each factor contributed to mental well‐being while controlling for the preceding variables.

The significance level was set at *p* < 0.05.

## RESULTS

### Participant demographics, work conditions, and psychological stress

#### Characteristics of the participants

Among participants, 58.4% were male, and the majority were in their 40s (39.7%). Most participants (87.9%) worked in private offices without colleagues. Of the participants, 21.4% had <3 years of experience as adult guardians, and 66.8% had <10 adult wards currently overseen. Their basic characteristics, professional backgrounds, and job conditions are summarized in Table 1.

**Table 1:** Basic characteristics of the participants.

	*n*	%
Basic characteristics	227	
Age		
30s and under	13	(5.8)
40s	89	(39.7)
50s	86	(38.4)
60s and over	36	(16.0)
Sex		
Male	129	(58.4)
Female	92	(41.6)
Workplace type		
Private office	197	(87.9)
Other	27	(12.1)
Professional background and job conditions		
Years of experience as adult guardian		
≤3 years	48	(21.4)
More than 4 years	176	(78.6)
Number of adult wards participants currently oversee		
≤10	141	(66.8)
≥11	70	(33.2)
Participant has experience working with adult wards diagnosed with		
Dementia	208	(99.5)
Schizophrenia	149	(72.0)
Intellectual disability	132	(62.9)
Percentage of time spent as an adult guardian in overall job	Mean ± SD	43.6 ± 26.2
The percentage of their total compensation derived from adult guardianship fee	Mean ± SD	36.0 ± 26.5

Abbreviations: *n*, number of participants; SD, standard deviation.

The effort–reward balance, calculated as (effort: the percentage of total working time devoted to adult guardianship)/(reward: the percentage of total compensation from guardianship fees), showed that 56.2% of participants exerted more effort than the reward they received (Table 2).

**Table 2:** Effort–reward balance.

Percentage of time spent as an adult guardian in overall job (Effort)			Percentage of compensation derived from adult guardianship fee (Reward)		
	*n*	%		*n*	%
～10	30	(14.5)	～10	55	(27.0)
～20	31	(15.0)	～20	28	(13.7)
～30	27	(13.0)	～30	28	(13.7)
～40	24	(11.6)	～40	16	(7.8)
～50	22	(10.6)	～50	22	(10.8)
～60	18	(8.7)	～60	16	(7.8)
～70	22	(10.6)	～70	20	(9.8)
～80	20	(9.7)	～80	10	(4.9)
～90	8	(3.9)	～90	4	(2.0)
～100	5	(2.4)	～100	5	(2.5)

Effort–reward balance		*n*	%		
Effort > Compensation		113	(56.2)		
Effort = Compensation		73	(36.3)		
Effort ＜ Compensation		15	(7.5)		

Abbreviation: *n*, number of participants.

#### Job perception, work conditions, and support

As shown in Table 3, 69.3% of participants reported being satisfied with serving as adult guardians. However, 58.4% considered their compensation inappropriate.

**Table 3:** Variables related to job and health.

Variables	*n*	%
Job perception, work conditions, and support		
Satisfaction with being an adult guardian		
Satisfied	149	(69.3)
Not satisfied	66	(30.7)
Perception of compensation for adult guardianship		
Appropriate	89	(41.6)
Not appropriate	125	(58.4)
Frequency of psychological stress		
High	88	(39.1)
Low	137	(60.9)
Main source of psychological stress		
Adult wards	40	(18.1)
Family members of adult wards	99	(44.8)
Staff of medical institutions	16	(7.2)
Staff of judicial institutions	11	(5)
Staff of administrative institutions	14	(6.3)
Other	38	(18.6)
Have job support		
Yes	196	(91.2)
No	19	(8.8)
Health‐related variables		
Self‐perceived physical health		
Good	190	(84.4)
Poor	35	(15.6)
Psychosocial variables		
Loneliness score (0–9)		
One‐point increase	Mean ± SD	4.0 ± 2.0
Burn out score (0–17)		
One‐point increase	Mean ± SD	6.9 ± 3.2
Community stigma score (12–48)		
One‐point increase	Mean ± SD	32.1 ± 4.9

Abbreviation: *n*, number of participants; SD, standard deviation

Regarding psychological stress, 39.1% of the participants reported experiencing frequent stress during their roles. The most common source of stress was interactions with family members of adult wards (44.8%), followed by stress related to the adult wards themselves (18.1%). Notably, 8.8% reported having no one to support them in guardianship tasks (Table 3).

#### Health‐related and psychosocial variables

In terms of physical health, 84.4% of the participants perceived themselves to be in good health. For psychosocial variables, the mean loneliness score (range: 0–9) was 4.0 (SD = 2.0), the mean burnout score (range: 0–17) was 6.9 (SD = 3.2), and the mean community stigma score (range: 12–48) was 32.1 (SD = 4.9) (Table 3).

### Distribution of mental well‐being

**Figure 1:**
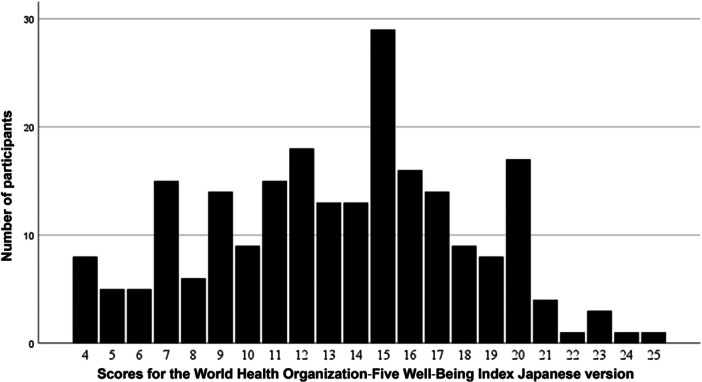
Distribution of mental well‐being according to participants’ scores for the World Health Organization‐Five Well‐Being Index Japanese version. The mean score ± SD was 13.37 ± 4.74. When a cut‐off criterion of 12/13 was used, the frequency of poor mental well‐being was 42.3%.

Investigation of the distribution of mental well‐being scores on the WHO‐5‐J was restricted to 226 participants (99.6%) with no missing values. The distribution of the total WHO‐5‐J scores is shown in Figure 1. The mean score ± SD was 13.37 ± 4.74. When a cut‐off criterion of 12/13 was used, the frequency of poor mental well‐being was 42.3%.

### Factors associated with mental well‐being

Univariate analysis identified several factors significantly associated with poor mental well‐being (Table 4), including low satisfaction with guardianship, frequent psychological stress, lack of job support, perceived poor physical health, high degree of loneliness, high level of burnout, and strong community stigma (Table 4).

**Table 4:** Factors associated with mental well‐being (univariate analysis).

Variables	*n*	*n* with poor mental well‐being (WHO‐5‐J < 13)	%	*β*	*t*	95% CI		*p* value	
Basic characteristics	227	96	42.3						
Age									
30s and under	13	9	(69.2)	0.08	1.16	−0.03	0.12	0.247	
40s	89	34	(38.2)						
50s	86	39	(45.3)						
60s and over	36	12	(33.3)						
Sex									
Male	129	58	(45.0)	0.04	0.56	−0.09	0.16	0.575	
Female	92	34	(37.0)						
Workplace type									
Private office	197	83	(42.1)	−0.02	−0.23	−0.22	0.18	0.821	
Other	27	12	(44.4)						
Professional background and job conditions									
Years of experience as adult guardian									
≤3 years	48	23	(47.9)	0.06	0.94	−0.08	0.24	0.348	
More than 4 years	176	71	(40.3)						
Number of adult wards participants currently oversee									
≤10	141	58	(41.1)	0.00	−0.04	−0.15	0.14	0.968	
≥11	70	29	(41.4)						
Participant has experience working with adult wards diagnosed with									
Dementia	208	89	(42.8)	0.08	1.18	0.00	0.01	0.238	
Schizophrenia	149	68	(45.6)	0.04	0.61	−0.01	0.02	0.541	
Intellectual disability	132	57	(43.2)	−0.06	−0.93	−0.02	0.01	0.353	
Percentage of time spent as an adult guardian in overall job									
	Mean ± SD	45.4 ± 26.9		−0.06	−0.84	0.00	0.00	0.400	
Percentage of participant's total compensation derived from adult guardianship fee	Mean ± SD	35.6 ± 25.9		0.01	0.20	0.00	0.00	0.845	
Job perception, work conditions and support									
Satisfaction with being an adult guardian									
Satisfied	149	49	(32.9)	0.27	4.15	0.15	0.43	<0.001	[**]
Not satisfied	66	41	(62.1)						
Perception of compensation for adult guardianship									
Appropriate	89	36	(40.4)	0.03	0.40	0.69	−0.11	0.163	
Frequency of psychological stress									
Not appropriate	125	54	(43.2)						
High	88	47	(53.4)	0.18	2.76	0.05	0.32	0.006	[*]
Have job support									
Low	137	48	(35.0)						
Yes	196	75	(38.3)	0.23	3.51	0.18	0.635	<0.001	[**]
No	19	15	(78.9)						
Health‐related variables									
Self‐perceived physical health									
Good	190	63	(33.2)	0.43	7.06	0.42	0.75	<0.001	[**]
Poor	35	32	(91.4)						
Psychosocial variables									
Loneliness score (0–9)									
One‐point increase	Mean ± SD	4.7 ± 1.9		−0.31	−4.68	−0.11	−0.044	<0.001	[**]
Burn out score (0–17)									
One‐point increase	Mean ± SD	8.4 ± 3.5		−0.39	−6.13	−0.08	−0.041	<0.001	[**]
Community stigma score (12–48)									
One‐point increase	Mean ± SD	32.9 ± 4.7		−0.15	−2.15	−0.03	−0.001	0.033	[*]

Abbreviations: *n*, number of participants; 95% CI, 95% confidence interval; SD, standard deviation; WHO‐5‐J, Japanese version of the World Health Organization‐Five Well‐Being Index.

* *p* < 0.05

** *p* < 0.001.

The results of the hierarchical multivariate analysis exploring the factors associated with mental well‐being are presented in Table 5. In the final model, frequent psychological stress (95% CI: 0.08–0.37, *p* = 0.002) and strong community stigma (95% CI: −0.03 to 0.00, *p* = 0.043) were independently associated with poor mental well‐being.

**Table 5:** Factors associated with mental well‐being of adult guardians (multivariate analysis).

	Model 1					Model 2					Model 3					Model 4				
	*β*	*t*		95% CI	*p*‐value	*β*	*t*		95% CI	*p*‐value	*β*	*t*		95% CI	*p* value	*β*	*t*	95% CI		*p* value
Basic characteristics																				
Sex	0.08	1.19	−0.05	0.21	0.236	0.11	1.62	−0.02	0.24	0.107	0.08	1.24	−0.05	0.22	0.218	0.12	1.71	−0.02	0.26	0.090
Years of experience as adult guardian ≤3 years	0.07	1.07	−0.07	0.25	0.285	0.07	1.05	−0.07	0.24	0.297	0.11	1.56	−0.03	0.29	0.120	0.10	1.41	−0.05	0.28	0.161
Subjective job strain																				
Frequency of psychological stress						0.21	3.15	0.08	0.35	0.002**	0.23	3.32	0.10	0.37	0.001**	0.22	3.10	0.08	0.37	0.002**
Reward‐related factor																				
Perception of compensation for adult guardianship											−0.05	−0.76	−0.19	0.08	0.447	−0.03	−0.44	−0.17	0.11	0.664
Psychosocial influence																				
Community stigma																−0.14	−2.04	−0.03	0.00	0.043*

Abbreviations: *n*, number of participants; 95% CI, 95% confidence interval.

* *p* < 0.05

** *p* < 0.001.

## DISCUSSION

Our clinical experience suggests that professionals working in guardianship systems frequently experience high levels of stress. Using the WHO‐5 as a measure of mental well‐being in working‐age populations, Gao et al.^22^ conducted a 2012 survey of 2796 employees in China and found that 34.9% reported poor mental well‐being, defined as a WHO‐5 score below 13. Similarly, analyzing data from the 2010 European Working Conditions Survey involving 33,443 employees across 34 European countries, Schütte et al.^23^ reported rates of poor mental well‐being at 28.3% among women and 23.6% among men. In comparison, our finding of a 42.3% prevalence of poor mental well‐being among professional guardians indicates a considerably higher rate than those observed in general working populations.

While extensive research has examined the mental well‐being of healthcare professionals globally,^24^, ^25^ limited studies have focused on the well‐being of professional guardians. Burnout among professionals in interpersonal support roles is well‐documented, with consequences including diminished quality of care and negative personal and organizational outcomes.^25^, ^26^, ^27^ Although guardianship responsibilities often extend beyond legal mandates,^28^ and maintaining good mental health is critical for responsible decision‐making,^8^ there is limited empirical evidence on the mental well‐being of professionals advocating for individuals with mental health conditions.

The study by Keul et al.^8^ of 134 professional guardians found that although participants did not report excessive mental distress overall, they exhibited notably high levels of phobic anxiety. Additionally, Chen and Gorski's interviews^29^ with 22 social justice and human rights activists highlighted burnout as a significant concern, emphasizing how a “culture of martyrdom” in rights advocacy increases the risk of mental health issues and underscores the need for greater societal support to sustain effective advocacy efforts.

Our findings revealed that 69.3% of the participants were satisfied with their role as adult guardians, but 58.4% were dissatisfied with their compensation. Additionally, 39.1% reported frequent psychological stress, with family members in adult wards identified as the primary source.

As noted, 83% of the participants were working in private offices without colleagues. We considered that this might negatively influence their mental well‐being; however, our analysis did not show a significant association between working alone and poor mental well‐being. One possible explanation is the limited sample size, which may have reduced the power to detect such effects.

Additionally, whether participants worked in private offices or not was not significantly related to perceived job support (95% CI: 0.54–5.03, *p* = 0.376) or loneliness (95% CI: 0.78–1.18, *p* = 0.672). Notably, 60.4% of those working in private offices and 51.9% of those working in other settings reported receiving job support from the Adult Guardianship Center Legal Support. This suggests that institutional support from the Center may serve as a key source of social and professional support, potentially mitigating the impact of working alone.

This study also found that frequent psychological stress and community stigma were independently associated with poor mental well‐being among professional guardians. Few studies have explored these factors in this context. Keul et al.^8^ examined personality traits and social competencies and reported that compulsiveness and defensiveness were correlated with poor mental well‐being, whereas self‐governance was correlated with better mental health. Our study is among the first to identify key factors associated with the mental well‐being of professional guardians.

The finding that community stigma significantly affects the well‐being of rights defenders offers new insights. The scale used in this study was originally designed to measure public stigma by assessing how respondents believed society viewed individuals with mental health conditions. Over time, it has also been applied to measure self‐stigma. Link^20^ observed that individuals with mental health conditions often internalize negative societal attitudes, affecting their self‐evaluation. We adapted the scale to assess guardians' perceptions of community stigma toward persons with mental health conditions.

Given that the top three reasons for the initiation of adult guardianship in Japan are dementia (62.6%), intellectual disability (9.9%), and schizophrenia (8.8%),^11^ guardians may be at risk of “stigma by association.” Goffman^30^ introduced this concept to describe how societal stigma extends beyond individuals with stigmatized conditions to those closely connected to them, such as family members, caregivers, and advocates. Research has indicated that stigma by association negatively affects self‐perception and interpersonal relationships, leading to increased psychological distress and reduced perceived closeness.^31^ Furthermore, it mediates the relationship between perceived public stigma and psychological distress, emphasizing its significant impact on mental well‐being.^31^ The broader consequences of stigma on well‐being are well documented,^32^ reinforcing the need for strategies that mitigate its effects.

One potential approach professionals in the field of psychiatry can take is to continue efforts to reduce stigma associated with mental health conditions. Although a variety of intervention strategies have been proposed,^33^ no single approach has proven definitively effective. While insufficient knowledge often contributes to stigma, increased knowledge alone does not necessarily reduce it.^34^, ^35^ Social contact has been suggested as an important mechanism for stigma reduction.^36^ For example, our previous study revealed that greater knowledge about dementia has been shown to reduce stigma toward persons living with dementia, but this effect is mostly limited to the mild stages of the condition. In later stages, meaningful social contact becomes more crucial in reducing stigma.^37^ However, caution is needed, as unstructured or poorly guided contact may inadvertently reinforce negative stereotypes.^32^ In our study, some professional guardians explained that, through their experiences, they came to understand that adult wards are supported by a network of professionals, including medical personnel. This realization helped reduce their own stigma toward individuals with mental health conditions. To prevent unintended consequences of contact, it is important that such interactions are structured and guided by professionals with expertise in psychiatry.

Although extensive research has explored the psychological burden of family caregiving on individuals with mental health conditions,^38^ the challenges faced by professional guardians remain underexplored. The Canadian Public Health Association^39^ emphasized that caregivers of individuals with mental health conditions often experience lower levels of subjective well‐being because of a lack of societal recognition and support. Historically, the mental health of caregivers has been undervalued, leaving them with few resources to effectively manage their responsibilities.

The findings suggest that the mental well‐being of rights defenders, including adult guardians, may be underestimated. Given the effectiveness of social support,^38^ such as peer support groups, in fostering social connectedness among individuals with shared experiences,^40^ establishing similar support systems for professional guardians can help address their unique challenges and improve well‐being.

The limitations of this study include its focus on guardians in Tokyo and relatively low response rate, which may limit its generalizability. Tokyo is a highly urbanized area with a well‐developed guardianship infrastructure and relatively abundant access to legal, medical, and social resources. As a result, professional guardians in Tokyo may experience different levels of institutional support, community awareness, and inter‐professional collaboration compared to those in rural or less‐resourced regions. This context could potentially lead to an underestimation or overestimation of certain findings. For example, the relatively high access to support services in Tokyo may mitigate psychological stress or loneliness, while urban environments might simultaneously increase caseload complexity or bureaucratic demands. Therefore, regional variations in system design, resources, and population characteristics could influence both the prevalence and the factors associated with mental well‐being among professional guardians.

Expanding the research to a broader population and incorporating qualitative insights are essential for developing comprehensive support strategies.

## CONCLUSION

This study highlights the significant mental health challenges faced by professional guardians in Japan, particularly the impacts of frequent psychological stress and community stigma. With 42.3% of the participants reporting poor mental well‐being, which is considerably higher than the general population, these findings underscore the urgent need for support mechanisms tailored to professional guardians.

Given the increasing reliance on professional guardians and their complex responsibilities, targeted interventions, such as peer support networks, stress‐management programs, and societal awareness initiatives, may help mitigate the psychological burden associated with their roles. Future research should further explore these issues through qualitative methods, such as in‐depth interviews, to gain deeper insight into the lived experiences of guardians and identify effective strategies to improve their well‐being.

## AUTHOR CONTRIBUTIONS

Kae Ito contributed to study design, data collection, analysis, drafting of the manuscript, and funding acquisition. Tsuyoshi Okamura contributed to data analysis and critically revised the manuscript for important intellectual content. Both authors have given final approval of the manuscript.

## CONFLICT OF INTEREST STATEMENT

The authors declare no conflicts of interest.

## ETHICS APPROVAL STATEMENT

All methods and procedures used in this study complied with relevant guidelines and regulations. Ethical approval was granted by the Institutional Review Board and Ethics Committee of the Tokyo Metropolitan Institute for Geriatrics and Gerontology (Approval No. R24‐058).

## PATIENT CONSENT STATEMENT

The online survey was conducted after participants were provided with a written explanation of the study's objectives, and informed consent was obtained electronically.

## CLINICAL TRIAL REGISTRATION

N/A.

## Data Availability

The data that support the findings of this study are available in the figshare database at https://figshare.com/, reference number 10.6084/m9.figshare.28379024. (https://doi.org/10.6084/m9.figshare.28379024).
